# A high plane of nutrition during early life alters the hypothalamic transcriptome of heifer calves

**DOI:** 10.1038/s41598-021-93080-4

**Published:** 2021-07-07

**Authors:** José M. Sánchez, Kate Keogh, Alan K. Kelly, Colin J. Byrne, Pat Lonergan, David A. Kenny

**Affiliations:** 1 School of Agriculture and Food Science, University College Dublin, Belfield, Dublin 4, Ireland; 2 Instituto Nacional de Investigación Y Tecnología Agraria Y Alimentaria, Ctr. de la Coruña Km 5.9, 28040 Madrid, Spain; 3 Teagasc Animal and Grassland Research and Innovation Centre, Grange, Dunsany, Co. Meath Ireland

**Keywords:** RNA sequencing, Transcriptomics

## Abstract

The aim was to examine the effect of rapid body weight gain during early calfhood consistent with earlier sexual development on the transcriptional profile of the hypothalamus. Angus X Holstein–Friesian heifer calves (19 ± 5 days of age) were offered a high (HI, n = 14) or moderate (MOD, n = 15) plane of nutrition from 3 to 21 weeks of age to achieve a growth rate of 1.2 kg/d and 0.5 kg/d, respectively. Following euthanasia at 21 weeks, the arcuate nucleus (ARC) region was separated from the remainder of the hypothalamus and both were subjected to RNA-Seq. HI calves exhibited altered expression of 80 and 39 transcripts in the ARC and the remaining hypothalamus, respectively (*P* < 0.05) including downregulation of *AGRP* and *NPY* and upregulation of *POMC*, previously implicated in precocious sexual development. Stress-signaling pathways were amongst the most highly dysregulated. Organ morphology, reproductive system development and function, and developmental disorder were amongst the networks derived from differentially expressed genes (DEGs) in the ARC. Gene co-expression analysis revealed DEGs within the ARC (*POMC*, *CBLN2*, *CHGA*) and hypothalamus (*PENK*) as hub genes. In conclusion, enhanced nutrition during early calfhood alters the biochemical regulation of the hypothalamus consistent with advanced sexual development in the prepubertal heifer.

## Introduction

Early onset of puberty is a key trait underpinning economically efficient cattle production systems. The recommended age of first calving, from both a biological and economical perspective, is 23 to 25 months^1–3^. Boulton et al.^3^ estimated that the mean daily cost of rearing a dairy heifer in the UK was £2.31 ± 0.41 (mean ± SD) with a predicted increase in mean cost of rearing of £2.87 for each extra day of age at first calving. To achieve this targeted calving age, however, puberty should be reached by 13 months of age^4^, since there is an increase in fertility (21%) from the first (pubertal) to the third postpubertal oestrus^5–7^.

In females, puberty can be defined as the first ovulatory oestrus followed by a normal luteal phase^8^. In cattle, puberty onset and the process of sexual maturation occurs in a gradual fashion regulated by a complex network of biochemical processes and involving interaction amongst many key metabolic, neuroendocrine and reproductive tissues ultimately culminating in maturation of the hypothalamic-hypophyseal-ovarian axis^9–11^. The hypothalamus is recognized as the homeostatic regulator of the body^12^. Although multiple hypothalamic areas are involved in the pathways mediating the metabolic regulation of neuroendocrine functions, major metabolic-sensing neurons are located within the arcuate nucleus (ARC). Signals from hormones associated with metabolic status such as leptin, ghrelin, insulin and insulin-like growth factor 1 (IGF-1) are integrated in the ARC through intermediate neuronal (kisspeptin neurons) and glial circuits that regulate gonadotropin-releasing hormone (GnRH) pulse generation (reviewed by Cardoso et al.^13^). Another population of hypothalamic kisspeptin neurons is localized in the preoptic area (POA) in ruminants and evidence suggests that they play a pivotal role in mediating positive feedback action of oestrogen to induce the GnRH/luteinizing hormone (LH) surge (reviewed by Uenoyama et al.^14^). Particularly in heifers, it has been reported that kisspeptin neuronal populations in the ARC and POA likely play important roles in regulating the GnRH pulse and surge, respectively^15^ and, therefore, are key regulators during the pubertal transition. The neuropeptide Y/agouti-related peptide (NPY/AGRP) and the proopiomelanocortin/cocaine- and amphetamine-regulated transcript (POMC/CART) neurons are two different neuronal populations also located in the ARC that contain the leptin receptor and that regulate the function of GnRH neurons (reviewed by Cardoso et al.^13^), highlighting the pivotal role of the hypothalamus and, specifically, the ARC in mediating the effect of nutrition on reproduction.

While heifers reach puberty at a breed-dependent genetically-influenced size^16, 17^, nutritional management during both the pre- and post-weaning period is the overarching factor that influences age at puberty^4, 11^. A restricted plane of nutrition during the prepubertal period delayed puberty in heifers^18, 19^ while elevated body weight (BW) gain during the same period advanced puberty due to an early maturation of the reproductive neuroendocrine system^20, 21^. Thus, age at puberty onset is inversely related to plane of nutrition and metabolic status, but this relationship is apparently more potent the earlier in life dietary augmentation is implemented^10^.

Most research evaluating the impact of nutrition on age at puberty in suckled beef heifers has focused on nutritional changes after weaning^19, 22^, with limited opportunity to manipulate early life nutritional status whilst suckling (without early-weaning). There is, however, a growing body of evidence supporting the importance of early life nutrition (first 6 months of life) and improved metabolic status during critical developmental windows early in juvenile development in regulating the timing of puberty in heifers by advancing maturation of the hypothalamus-pituitary–gonadal (HPG) axis^23–25^. Further, there is scarce knowledge on the molecular events associated with the nutritional imprinting of the hypothalamus during this early stage of development that regulate age at onset of puberty^24^.

Recent advances in deep-sequencing technology (RNA-Seq) provide the opportunity for in-depth insights into the global transcriptome of key biologically important tissues such as the hypothalamus, and, particularly, the ARC region as it is central to the interaction between metabolic signals and the neuroendocrine control of gonadotropin pulsatility. This will facilitate a better understanding of the biochemical and molecular interplay that conditions and eventually triggers the pubertal process in heifers and improvement of rearing protocols for heifer calves that ensure more efficient cattle production. Thus, the aim of this study was to examine the effect of enhanced nutrition during the first 21 weeks of life on the transcriptome of the ARC and the remaining hypothalamic tissue in juvenile heifer calves using RNA-Seq. We hypothesized that rapid BW gain during early calfhood, associated with an advancement in the age of puberty onset, would affect the transcriptional profile of hypothalamic tissue, and in particular, would elicit differential expression of biochemical pathways consistent with earlier sexual development.

## Results

### Early life growth

All details regarding pre- and post-weaning growth and energy intake of the calves involved in this study have previously been reported by Kelly et al.^26^. Overall, mean (± SD) BW of HI and MOD calves at slaughter at 21 weeks of age was 187.60 ± 4.62 and 117.73 ± 3.30 kg, respectively (*P* < 0.001). This translated into an average daily gain over the first 21 weeks of 1.18 kg/d and 0.50 kg/d for the HI and MOD dietary treatments, respectively (*P* < 0.001).

### Effect of plane of nutrition during early calfhood on transcriptional changes in the arcuate nucleus and remaining hypothalamic tissue

**Figure 1:**
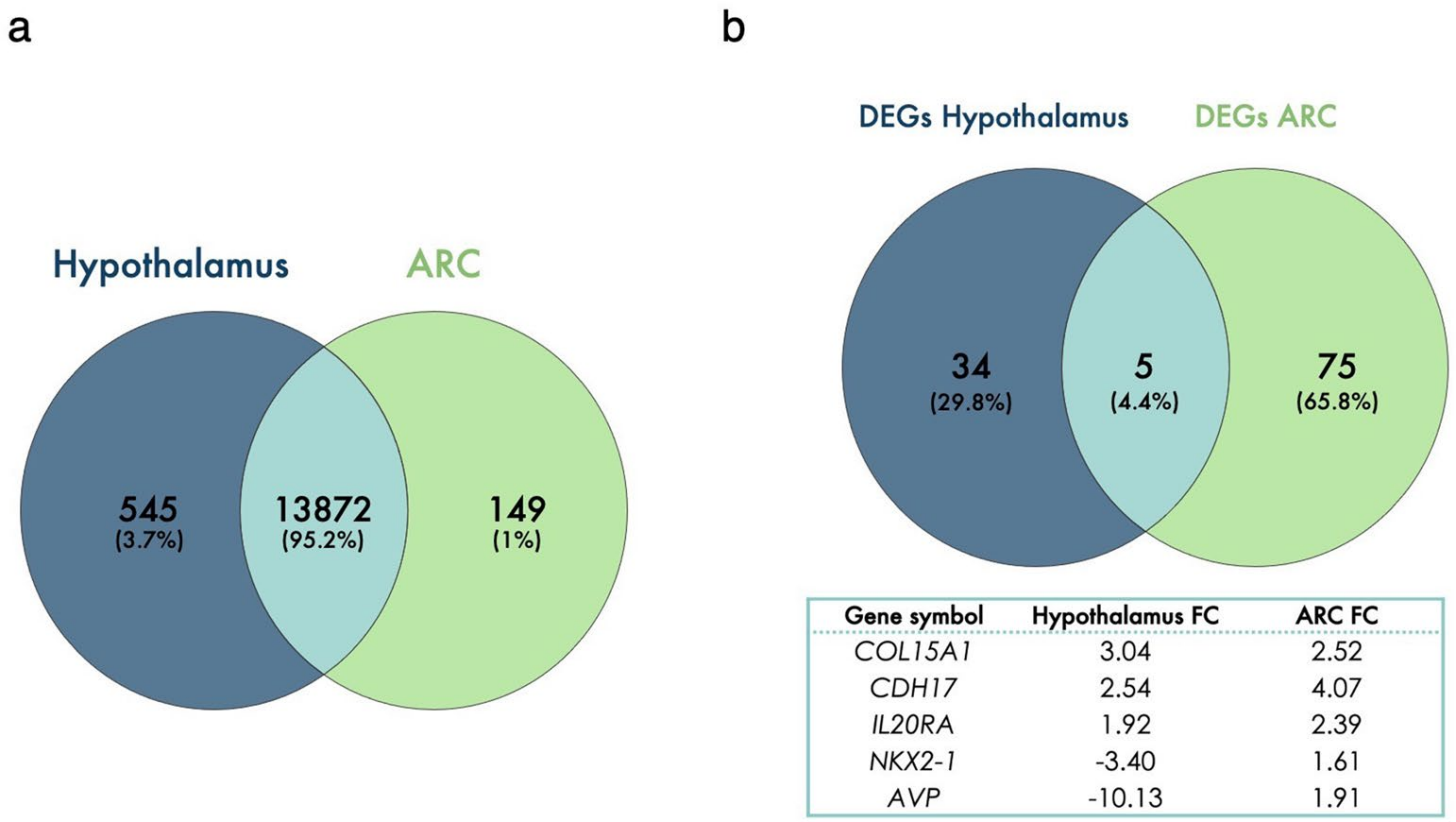
Venn diagram illustrating: (**a**) the variation in genes expressed across both the arcuate nucleus (ARC) and the remainder of the hypothalamus from all heifer calves, irrespective of their nutritional plane; (**b**) the number of differentially expressed genes (DEGs) in the ARC and the remainder of the hypothalamus of heifer calves on different nutritional planes. The solid lined box lists the transcripts common to both regions and their fold change (FC).

RNA sequencing of ARC and the remaining hypothalamic tissue samples generated on average 50 and 48 million reads per sample, respectively. Raw read counts, the number of reads obtained after quality control and mapping rates to the reference genome of individual samples, are listed in Supplemental Table S1A–B. Following alignment of trimmed sequencing reads to the bovine genome, on average more than 90% of the generated sequencing reads, across both tissue types, aligned to the protein coding regions of the genome, with 88% mapping rate identified as the lowest mapping percentage across all samples. Through the removal of lowly expressed genes within EdgeR analysis, 14,417 and 14,021 genes remained for differential expression analysis for ARC and remaining hypothalamic tissues, respectively. Of those, 13,872 genes (95.2%) were expressed in both tissues while 545 (3.7%) and 149 (1%) were uniquely expressed in ARC and remaining hypothalamus, respectively (Fig. 1a). RNA-Seq data derived from the current study have been deposited within NCBI’s Gene Expression Omnibus and are available through IDs GSE153495 and GSE153498 for hypothalamus and arcuate nucleus datasets, respectively.

**Figure 2:**
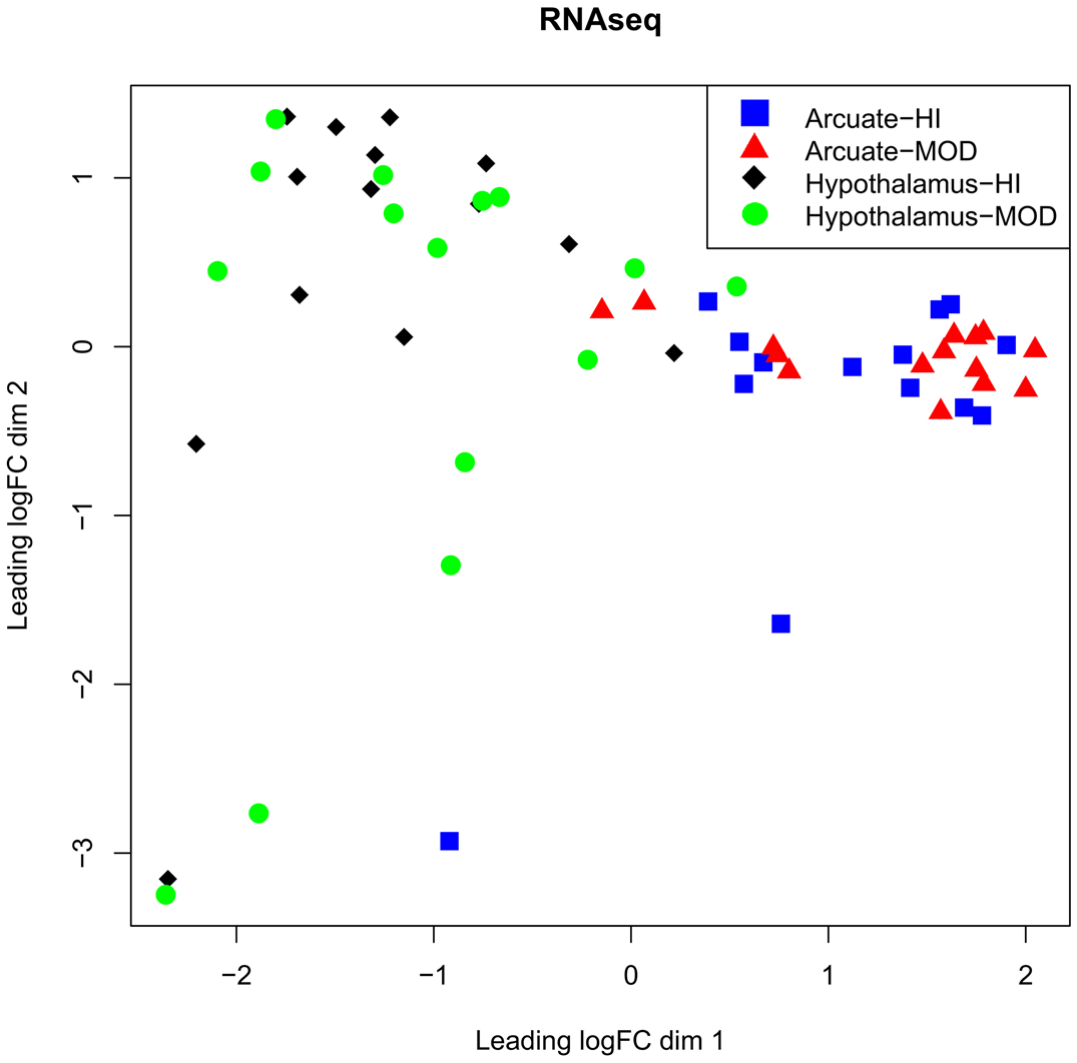
Multi-dimensional scaling plots for arcuate nucleus and the remainder of the hypothalamus of heifer calves on high (HI) vs moderate planes (MOD) of nutrition.

The biological coefficient of variation of samples is presented through multi-dimensional scaling plots for ARC and remaining hypothalamic tissue in Fig. 2. Data from both tissue samples cluster together irrespective of the prevailing plane of nutrition to which the calf was exposed.

### Diet-induced differentially expressed genes in the arcuate nucleus

The HI calves exhibited 80 DEGs (54 up- and 26 downregulated) compared to MOD contemporaries (q < 0.05; Supplemental Table S2. A high plane of nutrition reduced major histocompatibility complex, class II, DQ beta 1 (*HLA-DQB1*), *AGRP*, C3 and PZP like, alpha-2-macroglobulin domain containing 8 (*CPAMD8*) and *NPY* mRNA expression and increased glycoprotein hormones, alpha polypeptide (*CGA*), myosin light chain 2 (*MYL2*), cadherin 17 (*CDH17*), interleukin 20 receptor subunit alpha (*IL20RA*), and *POMC* mRNA expression in the ARC, amongst others (Table 1 and Supplemental Table S2). Interestingly, only five diet-induced DEGs were observed to be common to both the ARC and the remaining hypothalamic tissue (Fig. 1b): collagen type XV alpha 1 chain (*COL15A1*), *CDH17*, *IL20RA*, *NKX2-1*, and *AVP*. While *NKX2-1* and *AVP* were upregulated in the ARC of HI calves, those transcripts were amongst the most downregulated in the remaining hypothalamic tissue of the same animals. In contrast, *COL15A1*, *CDH17*, and *IL20RA* were upregulated in both hypothalamic regions of HI calves (Fig. 1b).

**Table 1:** Gene ID, gene symbol, gene name, and fold change of most upregulated and downregulated (fold change > 2) differentially expressed genes (DEGs) in the arcuate nucleus of prepubertal heifers on high vs moderate planes of nutrition (q < 0.05).

Gene ID	Gene symbol	Gene name	Fold change
ENSBTAG00000016138	*CGA*	Glycoprotein hormones, alpha polypeptide	11.95
ENSBTAG00000018369	*MYL2*	Myosin light chain 2	4.45
ENSBTAG00000021964	*CDH17*	Cadherin 17	4.07
ENSBTAG00000007131	*GADL1*	Glutamate decarboxylase like 1	2.73
ENSBTAG00000010082	*COL15A1*	Collagen type XV alpha 1 chain	2.52
ENSBTAG00000015638	*IL20RA*	Interleukin 20 receptor subunit alpha	2.39
ENSBTAG00000021251	*RIPK4*	Receptor interacting serine/threonine kinase 4	2.33
ENSBTAG00000013352	*NKX2-8*	NK2 homeobox 8	2.32
ENSBTAG00000010875	*MSX1*	msh homeobox 1	2.29
ENSBTAG00000038748	*HBD*	Hemoglobin subunit delta	2.23
ENSBTAG00000019455	*MYO5B*	Myosin VB	2.21
ENSBTAG00000017174	*SCN11A*	Sodium voltage-gated channel alpha subunit 11	2.20
ENSBTAG00000002644	*KCNQ4*	potassium voltage-gated channel subfamily Q member 4	2.20
ENSBTAG00000005525	*LHX6*	LIM homeobox 6	−2.29
ENSBTAG00000038128	*HLA-DQA2*	major histocompatibility complex, class II, DQ alpha 2	−2.41
ENSBTAG00000009331	*CPAMD8*	C3 and PZP like, alpha-2-macroglobulin domain containing 8	−2.42
ENSBTAG00000014556	*AGRP*	Agouti related neuropeptide	−2.92
ENSBTAG00000021077	*HLA-DQB1*	Major histocompatibility complex, class II, DQ beta 1	−4.65

For full list of DEGs, see Supplemental Table S2.

**Figure 3:**
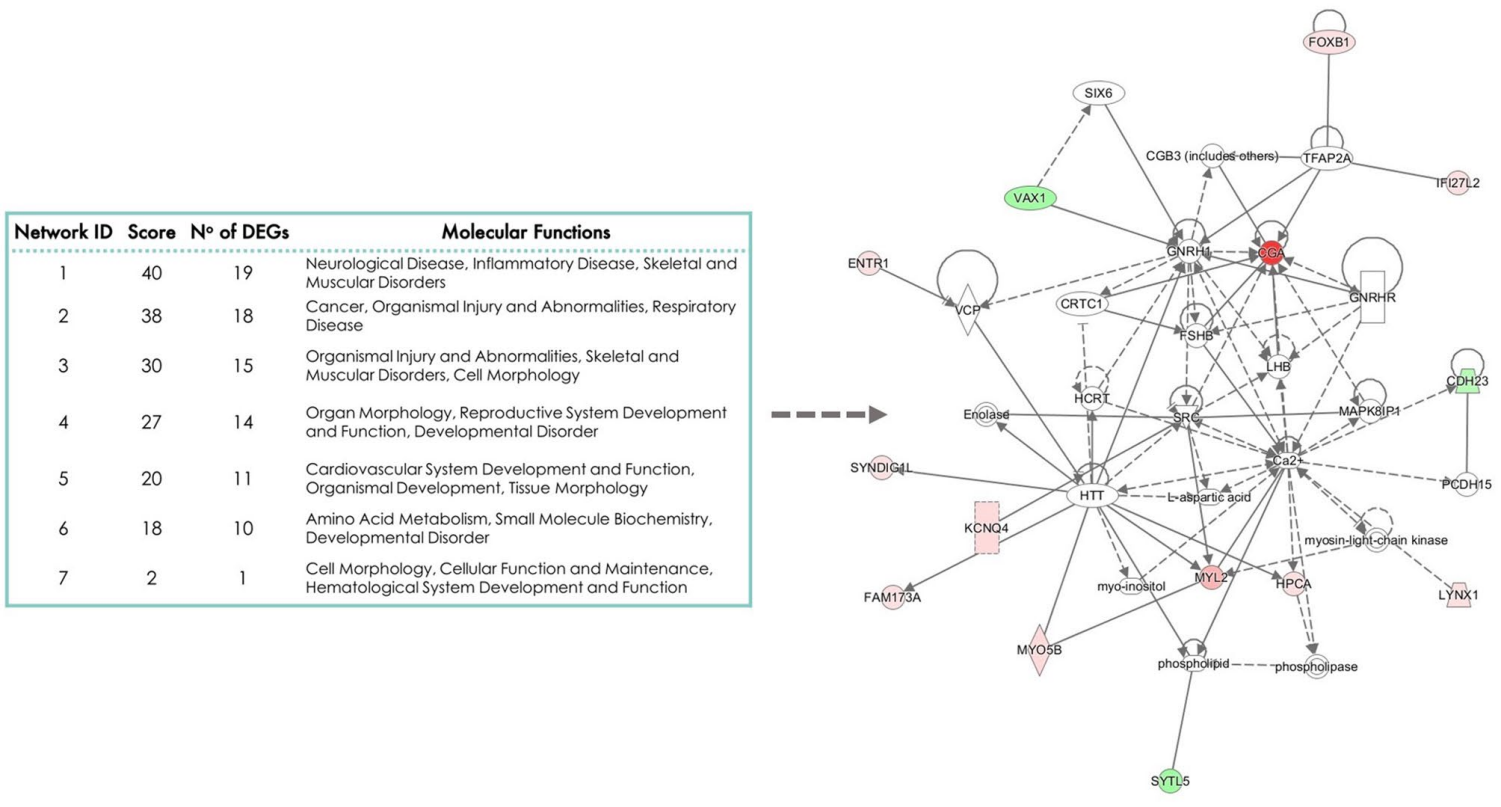
Networks derived from the list of differentially expressed genes (DEGs) in the arcuate nucleus (ARC) of prepubertal heifers on different planes of nutrition. Note that the higher the score the more connected the genes are. No of DEGs = number of DEGs from the input dataset included in the network. The arrow indicates Network 4: organ morphology, reproductive system development and function, developmental disorder derived from the list of DEGs in the ARC. Note that 14 of the DEGs in the ARC are included in this network (downregulated in green and upregulated in pink-red).

Within ARC tissue, IPA revealed 38 pathways enriched by DEGs identified between the two dietary treatment groups (Supplemental Table S3). The top 10 most overrepresented pathways in the ARC were antigen presentation pathway, autoimmune thyroid disease signaling, allograft rejection signaling, B Cell development, OX40 signaling pathway, Cdc42 signaling, graft-versus-host disease signaling, Type I Diabetes Mellitus signaling, neuroinflammation signaling pathway, and Nur77 signaling in T Lymphocytes. Furthermore, functional processes and DEGs involved in each process are listed in Supplemental Table S4. The top 10 functional processes found in the ARC were embryonic development, organismal development, behaviour, endocrine system development and function, lipid metabolism, molecular transport, small molecule biochemistry, cellular development, cellular growth and proliferation, and nervous system development and function. In addition, seven networks were derived from the list of DEGs in the ARC (Fig. 3) including organ morphology, reproductive system development and function, developmental disorder, with 14 of the DEGs in the ARC included in this network. Of note, *CGA*, the most upregulated DEG in ARC, was one of the most connected genes within this network (Fig. 3).

Upstream analysis revealed nine potential upstream regulators included in the list of DEGs that controlled the expression of other DEGs found in the ARC (Table 2A) with *CGA* (the most upregulated transcript in ARC) and *HLA-DQB1* (the most downregulated transcript) amongst them. In addition, brain-derived neurotrophic factor (*BDNF*), appetite-regulating hormone (*GHRL*), forkhead box O1 (*FOXO1*), estrogen receptor 1 (*ESR1*), and nuclear receptor subfamily 3 group C member 1 (*NR3C1*) were amongst those predicted to be most inhibitory upstream regulators by Z-score not included in the list of DEGs in this study and adenylate cyclase activating polypeptide 1 (*ADCYAP1*), leptin receptor, leptin, neuropeptide Y receptor Y2 (*NPY2R*), *TNF*, and interleukin 27 (*IL27*) amongst those predicted to be the most activated (Supplemental Table S5). Finally, chemicals such as L-triiodothyronine, progesterone, testosterone, corticosterone, dexamethasone, and glucocorticoid were also those predicted to be inhibitory upstream regulators while insulin, beta-oestradiol, L-Dopa, and calcitriol were predicted to be the activated upstream regulators (Supplemental Table S5).

**Table 2:** Upstream regulators controlling the expression of differentially expressed genes (DEGs) in the arcuate nucleus (A) and the remaining hypothalamic tissue (B) of heifer calves on high vs moderate planes of nutrition that are included in the list of those DEGs. TR = transcription regulator.

Upstream regulator	Fold change	Molecule type	*P*-value	Target
*(A)*				
*NPY*	−2.09	Other	0.000222	*AVP,NPY,POMC*
*CARTPT*	1.74	Other	0.000593	*AVP,CARTPT*
*AVP*	1.91	Other	0.000742	*AVP,EGR1,POMC*
*MSX1*	2.29	TR	0.0027	*CGA,MSX1*
*EGR1*	−1.73	TR	0.00509	*CARTPT,CHGA,EGR1,VCAM1*
*CGA*	11.95	Other	0.00583	*CGA,CLDN5*
*ISG15*	1.79	Other	0.0255	*MX1*
*HLA-DQB1*	−4.65	Other	0.0291	*HLA-DQA1*
*KMT2A*	−1.55	TR	0.0402	*EGR1,ZIC1*
*(B)*				
*GAL*	−1.84	Other	0.000003	*AVP,OXT,PRL*
*SCGN*	−4.52	Other	0.00175	*TAC1*
*TAC1*	−1.82	Other	0.00974	*PDYN,TAC1*
*CALCB*	−3.70	Other	0.0105	*PRL*
*PRL*	1.77	Cytokine	0.0126	*OXT,PENK,PRL*
*OXT*	−2.44	Other	0.0379	*MYH7*

### Diet-induced differentially expressed genes in the remaining hypothalamic tissue

Compared with calves on MOD, a HI plane of nutrition altered the expression of 39 (9 up- and 30 downregulated) transcripts in the remaining hypothalamus (q < 0.05; Supplemental Table S6). Orosomucoid 1 (*ORM1*), *COL15A1*, *CDH17*, cholinergic receptor nicotinic beta 3 subunit (*CHRNB3*) and anoctamin 3 (*ANO3*) were amongst the most upregulated transcripts in this hypothalamic tissue sections in HI calves (Table 3). Enhanced nutrition induced the downregulation of 30 DEGs in the remaining hypothalamus with arginine vasopressin (*AVP*), secretagogin, EF-hand calcium binding protein (*SCGN*), hypocretin neuropeptide precursor (*HCRT*), calcitonin related polypeptide beta (*CALCB*), and NK2 homeobox 1 (*NKX2-1*) amongst the most downregulated transcripts (Table 3).

**Table 3:** Gene ID, gene symbol, gene name, and fold change of most upregulated and downregulated (fold change > 2) differentially expressed genes (DEGs) in the remaining hypothalamic tissue of heifer calves on high vs moderate planes of nutrition (q < 0.05).

Gene ID	Gene symbol	Gene name	Fold change
ENSBTAG00000017294	*ORM1*	Orosomucoid 1	5.65
ENSBTAG00000010082	*COL15A1*	Collagen type XV alpha 1 chain	3.04
ENSBTAG00000021964	*CDH17*	Cadherin 17	2.54
ENSBTAG00000014050	*CHRNB3*	Cholinergic receptor nicotinic beta 3 subunit	2.40
ENSBTAG00000021855	*ANO3*	Anoctamin 3	2.18
ENSBTAG00000009336	*LXN*	Latexin	2.02
ENSBTAG00000020979	*NGFR*	Nerve growth factor receptor	−2.04
ENSBTAG00000005888	*MDGA1*	MAM domain containing glycosylphosphatidylinositol Anchor 1	−2.14
ENSBTAG00000008497	*RGS14*	Regulator of G protein signaling 14	−2.17
ENSBTAG00000031135	*CTXN3*	Cortexin 3	−2.19
ENSBTAG00000008319	*DUSP9*	Dual specificity phosphatase 9	−2.20
ENSBTAG00000014100	*BAIAP3*	BAI1 associated protein 3	−2.20
ENSBTAG00000009155	*SCN5A*	Sodium voltage-gated channel alpha subunit 5	−2.32
ENSBTAG00000003515	*SNCG*	Synuclein gamma	−2.40
ENSBTAG00000013548	*GABRE*	Gamma-aminobutyric acid type A receptor epsilon subunit	−2.42
ENSBTAG00000008026	*OXT*	Oxytocin/neurophysin I prepropeptide	−2.45
ENSBTAG00000000828	*CAPN6*	Calpain 6	−2.55
ENSBTAG00000006354	*HP*	Haptoglobin	−2.57
ENSBTAG00000009650	*OTOF*	Otoferlin	−2.77
ENSBTAG00000016766	*TMEM176B*	Transmembrane protein 176B	−2.81
ENSBTAG00000004924	*PENK*	Proenkephalin	−2.87
ENSBTAG00000000541	*NKX2-1*	NK2 homeobox 1	−3.41
ENSBTAG00000001516	*CALCB*	Calcitonin related polypeptide beta	−3.70
ENSBTAG00000000665	*HCRT*	Hypocretin neuropeptide precursor	−3.75
ENSBTAG00000019926	*SCGN*	Secretagogin, EF-hand calcium binding protein	−4.52
ENSBTAG00000008027	*AVP*	Arginine vasopressin	−10.13

For full list of DEGs, see Supplemental Table S6.

To understand the functional implication of DEGs found in the remaining hypothalamic tissue between HI and MOD calves, we performed pathway analysis. The most overrepresented pathways in the remaining hypothalamus were: acute phase response signalling, hepatic fibrosis/hepatic stellate cell activation, opioid signalling pathway, neuroprotective role of THOP1 in Alzheimer's disease, LXR/RXR Activation, STAT3 pathway, tight junction signalling, cardiomyocyte differentiation via BMP receptors, production of nitric oxide and reactive oxygen species in macrophages, and circadian rhythm signalling (Supplemental Table S7). In addition, the top 10 functional processes found in the remaining hypothalamus were: cell-to-cell signalling and interaction, molecular transport, small molecule biochemistry, behaviour, cell signalling, vitamin and mineral metabolism, nervous system development and function, nucleic acid metabolism, carbohydrate metabolism, and cellular function and maintenance. Full lists of functional processes and DEGs involved in each process are available in Supplemental Table S8. Finally, three networks were derived from the list of DEGs in this tissue, the biological functions of which were: behaviour, dermatological diseases and conditions, neurological disease (19 DEGs included within the network), cancer, organismal injury and abnormalities, respiratory disease (14 DEGs), and cell signalling, nucleic acid metabolism, small molecule biochemistry (6 DEGs).

Results from the conventional downstream analysis approaches described above give relevant insight into the potential effects of the induced alterations in gene expression. However, they provide only limited clues to the underlying causes that provoke such effects. Therefore, we also carried out an upstream analysis enabling a causal interpretation of the observed expression changes. Six potential upstream regulators, included in the list of DEGs, that controlled the expression of other DEGs (target genes) found in the remaining hypothalamus are listed in Table 2B. Of those, *SCGN*, *CALCB*, and oxytocin/neurophysin I prepropeptide (*OXT*) were amongst the most downregulated transcripts in the remainder of the hypothalamus of HI compared to MOD calves. In addition, excluding chemicals, the four top-most predicted inhibited upstream regulators by Z-score not included in the list of DEGs in this study were leukemia inhibitory factor (*LIF*), cAMP responsive element binding protein 1 (*CREB1*), interleukin 1 beta (*IL1B*), and tumor necrosis factor (*TNF*) and the two top-most predicted activated upstream regulators were potassium two pore domain channel subfamily K member 9 (*KCNK9*) and leptin (Supplemental Table S9). Interestingly, chemicals such as L-Dopa, dexamethasone, beta-oestradiol, and thyroid hormone were amongst the predicted to be the most inhibited upstream regulators while progesterone and testosterone were amongst the predicted to be the most activated upstream regulators (Supplemental Table S9).

### Weighted gene co-expression network analysis in the arcuate nucleus and the remaining hypothalamic tissue

Weighted gene co-expression network analysis was used to generate networks of co-expressed genes. Resultant networks of co-expressed genes were then mined for genes that have previously been implicated in puberty development in heifers^27, 28^ as well as genes identified as DEG within the current study, in order to determine the underlying biological interactions of genes associated with puberty development in heifers. Network analysis resulted in the generation of 15 networks of co-expressed genes in the ARC with 15 genes previously associated with puberty also identified as hub genes within our dataset, including: nebulette (*NEBL*, green network); *CBLN1*, *CBLN2*, *CBLN4*, neurotensin (*NTS*), *SCG2* and somatostatin (*SST*) in the magenta network; copine 5 (*CPNE5*) and protein phosphatase 3 catalytic subunit alpha (*PPP3CA*) in the pink network; inhibin subunit alpha (*INHA*), proprotein convertase subtilisin/kexin type 1 inhibitor (*PCSK1N*), proenkephalin (*PENK*), *POMC* and *SCG3* in the red network and tumor susceptibility 101 (*TSG101*) in the yellow network . A full list of target genes for each of these hub genes and GO results for these networks can be found in Supplemental Tables S10 and S11, respectively. Briefly, magenta network includes genes involved in biological processes including cellular response to oestradiol stimulus, axonogenesis and neuropeptide signalling pathway. Genes included in the pink network enrich pathways such as oxytocin, GnRH and calcium signalling pathways. Protein tyrosine phosphatase activity and GTPase activator activity were amongst the molecular functions associated with genes included in the red network in the ARC.

In the remaining hypothalamus, WGCNA resulted in the generation of 14 networks of co-expressed genes (each designated a separate colour name). Within these networks seven genes previously identified to be important to puberty or to harbour SNPs associated with puberty were identified as hub genes, controlling the expression of several other genes in the network. These included: cerebellin 1 precursor (*CBLN1*) and secretogranin II (*SCG2*) in the salmon network; cerebellin 2 precursor (*CBLN2*) and chromogranin B (*CHGB*) in the green network; cerebellin 4 precursor (*CBLN4*) in the greenyellow network; chromogranin A (*CHGA*) in the brown network and secretogranin III (*SCG3*) in the purple network. Target genes for each of these hub genes are listed in Supplemental Table S12. Of note, brown and green networks include genes involved in pathways including metabolic pathway and biosynthesis of amino acid. Full information of GO results for the networks that included hub genes listed above are outlined in Supplemental Table S13.

Interestingly, of those genes previously associated with puberty in cattle^27, 28^, *POMC* (upregulated)*, CBLN2* (downregulated), and *CHGA* (upregulated) were differentially expressed in the ARC in this study while *PENK* (downregulated) was amongst the DEGs in the remainder of the hypothalamus. Furthermore, these genes were also hub genes across both ARC and hypothalamic datasets in the current study.

## Discussion

Early life nutrition, especially during the first 6 months, is critical in regulating the timing of puberty in both male and female cattle by advancing maturation of HPG axis (reviewed by Kenny et al.^10^). However, how metabolic signal influence molecular changes in upstream circuitries within the hypothalamus to regulate this process remain unclear. Using an animal model known to accelerate puberty onset^26^ in which heifer calves were offered a high or moderate plane of nutrition from 3 to 21 weeks of age, we studied the diet-induced alterations, specifically, in the ARC and, more globally, in the remaining hypothalamic tissue. Main findings were that: (i) compared with calves on MOD, a high plane of nutrition altered the expression of 80 and 39 transcripts in the ARC and the remaining hypothalamic tissue, respectively; (ii) a distinct functional implication of DEGs in the ARC and the remaining hypothalamus related to different diets was found, with stress-signaling pathways amongst the most highly dysregulated pathways in both tissues; and, (iii) gene co-expression analysis resulted in the generation of 15 and 14 networks of co-expressed genes in the ARC and the remaining hypothalamus, respectively. Further, we identified DEGs from the current study as well as genes previously associated with puberty in cattle^27, 28^ as hub genes, regulating the expression of other genes within their respective networks. These included *POMC*, *CBLN2* and *PENK* within the ARC and *CHGA* within the remainder of the hypothalamus, further implicating a role for these genes in mediating metabolic status with subsequent reproductive development.

As we hypothesized, rapid BW gain during early calfhood (1.18 kg/d vs. 0.50 kg/d for the HI and MOD, respectively) altered the transcriptional profile of the hypothalamic tissue. It is well known that the onset of puberty in cattle is affected by nutritional management amongst other factors^4, 29^. In particular, metabolic status during the first 6 months of age is of critical importance in regulating the timing of puberty in cattle by advancing maturation of HPG axis^24, 25, 30, 31^. Moreover, early life nutritional programming has a lasting beneficial influence on a range of key economically important traits in cattle production. For example, an increased growth rate during the first 6 months of age can stimulate mammary development and improve the health and performance of dairy heifers^32, 33^. Thus, the results described in this manuscript are of significant value for understanding differential expression of biochemical pathways, consistent with earlier sexual maturation to optimise rearing systems for cattle.

We identified 80 and 39 diet-induced DEGs in the ARC and the remaining hypothalamic tissue, respectively. In particular, a high plane of nutrition reduced *AGRP* and *NPY* mRNA expression and increased *POMC* mRNA expression in the ARC. In agreement with our hypothesis, such expression patterns in the ARC of prepubertal heifers with increased BW gain between 4 and 8 months of age have recently been associated with increases in the pulsatile release of GnRH and LH and with early onset of puberty (reviewed by Cardoso et al.^13^). Both NPY and AGRP are two main orexigenic neuropeptides secreted during conditions of low energy balance by the NPY/AgRP neurons^34, 35^. In contrast, the *POMC* gene expressed in POMC/CART neurons encodes several peptides, including the anorexigenic alpha-melanocyte stimulating hormone (α-MSH), which is produced primarily during periods of positive energy balance^36^. These two neuronal populations located in the ARC directly regulate the function of GnRH neurons and, therefore, GnRH pulsatility and surge^37^. GnRH is also regulated by other afferent metabolic regulatory signals including leptin and its increased systemic concentration as a consequence of a high plane of nutrition has been associated with reduced *AGRP* and *NPY* mRNA expression and increased *POMC* mRNA expression in heifers^24, 25, 38^. However, we did not observe a difference between the contrasting planes of nutrition in the expression of the leptin receptor and/or *GNRH1* in either hypothalamic tissues. Notwithstanding this, however, leptin receptor was co-expressed with *GABARAP*, *GABRB3* (GABA receptors), *GNA11* (guanine nucleotide-binding protein subunit alpha-11), *INSR* (insulin receptor), and *SEMA5A* (semaphoring 5A) which are involved in GnRH signalling^39–42^, highlighting the relationship between metabolic status and reproductive development at this early stage. Upstream analysis revealed that leptin was one of the top ranking predicted activated upstream regulators in both the ARC and the remaining hypothalamic tissues. Interestingly, in associated studies from our group using the same calves, leptin concentrations were greater in the HI compared with MOD calves at 20 weeks of age (one week before the collection of hypothalamic samples) but not before^43^. Although Kelly et al.^26^ found differences in size of the anterior pituitary, concentrations of LH in response to a GnRH challenge (at 19 weeks of age) were not affected by early life diet. On the contrary, MOD calves had higher FSH concentrations than HI calves at 10, 15, and 20 weeks of age and also from 60 min through to 135 min after GnRH administration. Greater oestradiol output recorded in calves on the HI plane of nutrition was consistent with the observed greater ovarian surface follicle numbers and oocytes recovered at 21 weeks of age^26^. This decrease in FSH in HI calves could be due to an earlier shift towards gonad-dependent suppression of GnRH, which develops progressively during the juvenile period and reflects an increase in responsiveness to negative feedback from oestradiol^44^. Furthermore, plane of nutrition did not affect endometrial gland development at 21 weeks of age but altered reproductive organ growth and the endometrial transcriptome^45^. Of note, prepubertal endometrium was capable of responding to early embryos (Day 7 and 14) and interferon tau in a manner similar to that of the postpubertal endometrium^45^. Taken together, these results suggests that the functionality of each constituent organ within the HPG axis is affected by prevailing metabolic status though there seems to be some asynchrony between tissues in their relative rate of ontogenesis. Although the pituitary, ovaries and endometrium of prepubertal heifers at this age are capable of responding to stimuli in a manner similar to that of their postpubertal contemporaries, the essential maturation of the reproductive neuroendocrine system for the onset of puberty relies on the appropriate development of the hypothalamus. This concept is further supported by the observations of Cánovas et al.^46^ who reported that, amongst the various reproductive tissue studied (hypothalamus, pituitary, uterus-endometrium, and ovary), the hypothalamus experienced the most notable upregulation of genes upon onset of puberty.

Ingenuity pathway analysis revealed a distinct functional implication of DEGs in the ARC and the remaining hypothalamus in response to prevailing diet. Signaling by the metabolic hormones leptin and insulin is dynamically regulated by stress-signaling pathways to control feeding behavior and biosynthetic processes^47^. This is consistent with our results, as the observed differences in the concentrations of leptin, insulin, IGF-1, and glucose, induced by the contrasting planes of nutrition employed^26^, were associated with dysregulated acute phase response signaling in the remainder of the hypothalamus and antigen presentation pathway in the ARC. In addition, leptin was one of the top-most predicted activated upstream regulators in both hypothalamic regions. Such pathways are critical for normal cellular homeostasis and adaptive changes in cell physiology that benefit the organism^47^. Furthermore, the immune system possesses the ability to block normal reproductive functioning^48, 49^. At the central level, IL1B seems to play the most important role in the suppression of GnRH secretion during immune challenge^48^. Matthews et al.^49^ associated upregulation of *IL1B* with abrupt onset of anoestrus after a short term dietary restriction in heifers. In the current study, although *IL1B* was not differentially expressed, it was found amongst the top-most predicted inhibited upstream regulators in the remaining hypothalamus. It has been postulated that the inhibitory action of *IL1B* on GnRH may involve direct action on the GnRH neurons or indirect action involving other mediators such as opioids, catecholamines, gamma-aminobutyric acid, prostaglandins or nitric oxide (reviewed by Tomaszewska-Zaremba and Herman^48^). This indirect action is supported by our results as opioid signaling pathway and production of nitric oxide and reactive oxygen species in macrophages were amongst the most enriched pathways by DEGs in the remainder of the hypothalamus and L-Dopa amongst the most predicted inhibited upstream regulators.

Furthermore, we found five target genes for IL1B that were differentially expressed in the remaining hypothalamic tissue (*AVP*, *HP*, *NGFR*, *ORM1* and *TAC1*). Of those, only *AVP* and *TAC1* have been implicated in reproductive functions. Arginine vasopressin is the product of a clock-controlled gene that plays a crucial role in modulating the hypothalamus–pituitary–adrenal axis, which integrates the response to stress. It is mainly synthesized in the supraoptic nucleus and paraventricular nucleus of the hypothalamus^50, 51^. In this study, *AVP* was the most downregulated transcript in the remainder of the hypothalamus of HI compared with MOD calves but it was upregulated in the ARC. This contrasting expression pattern could be due to the presence of a number of tissue-specific elements that participate in the differential regulation of vasopressin transcription observed in different brain regions^52^. This gene is part of the circadian rhythm pathway, which was one of the top 10 enriched pathways by DEGs in the remaining hypothalamic tissue of HI calves in our study. The circadian rhythm system is controlled by the transcription of circadian clock genes and regulates several physiological reproductive processes in mammals including ovarian function, responsiveness to gonadotropins and ovulation^53^.

It has recently been reported that the kisspeptin neuronal populations in the ARC and POA likely play important roles in regulating the GnRH pulse and surge, respectively, in heifers^15^. These authors also observed that both neurokinin B (NKB) and dynorphin A (Dyn) were only co-localized in kisspeptin neurons in the ARC, demonstrating the presence of kisspeptin/NKB/Dyn-containing neurons (referred to as KNDy neurons), but not in the POA. Neurokinin B, together with Substance P (SP) and neurokinin A (NKA), belong to a family of peptides termed tachykinins while Dyn is an endogenous opioid peptide. LH pulses are positively or negatively regulated by NKB or Dyn, respectively, in rodents^54^ and goats^55^, highlighting their importance in the regulation of GnRH pulsatility. Interestingly, Dyn, is encoded by *PDYN,* which was downregulated in the remainder of the hypothalamus of HI compared with MOD calves. Moreover, *PDYN* was co-expressed in a network with *NPYR* in the ARC, with *NPYR* regulating the expression of *PDYN*. Additionally *SEMA3G* which encodes a semaphorin gene, involved in GnRH system development was also included within this network. In contrast to NKB and Dyn, there is no information about the role of *TAC1*-encoded peptides (SP and NKA) in the control of GnRH release during the prepubertal period in cattle. Substance P has been associated with pain perception and inflammatory processes in the brain^56^. In a recent study, Navarro et al.^57^ identified a potent regulation of gonadotropin release by the SP and NKA and their respective receptors in the presence of kisspeptin-Kiss 1 receptor signaling in mice. These authors also reported that *Tac1* expression in the ARC and ventromedial nucleus was inhibited by circulating oestradiol. We did not observe dietary-induced differences in the expression of *TAC1* in the ARC but it was downregulated in the remainder of the hypothalamus tissue of HI compared with MOD calves. However we did observe for *TAC1* to be co-expressed in a network within the ARC involved in endoplasmic reticulum and neuronal cell body, moreover in this network *TAC1* was interacting with *SIRT3* (sirtuin 3), which is central to regulating metabolic status. In addition, upstream analysis showed that beta-oestradiol was amongst the most predicted inhibited upstream regulators in the remaining hypothalamus while, on the contrary, it was amongst the most predicted activated upstream regulator in the ARC. Previously, we have also shown that HI heifers had higher concentrations of oestradiol than MOD calves^26^. There is strong evidence that oestradiol reflects an inhibition of kisspeptin release from KNDy neurons, and stimulation of Dyn release, that wanes with the decrease in the negative feedback actions of oestradiol during puberty in ewes (reviewed by Nestor et al.^58^). As stated above, in the present study, *PDYN* expression was downregulated. These results suggest a distinct sensitivity to oestradiol in different regions of the hypothalamus during the prepubertal period in cattle that could be influenced by prevailing nutritional status. These differences could affect kisspeptin biosynthesis and secretion and therefore, the timing of pubertal onset. There is relatively little information about the role of *TAC1*-encoded peptides (SP and NKA) in the control of GnRH release during the prepubertal period in cattle and their regulation by oestradiol, and therefore this area warrants further research.

An alternative reason for the dysregulation of signaling pathways related to inflammation and immune response in the hypothalamus following distinct nutritional management in prepubertal calves is: (i) to facilitate a neuroprotective effect; or (ii) to aid in the remodelling and/or development of the hypothalamus during the prepubertal period. Energy deficit caused by diet restriction may induce the expression of immune system genes to protect the brain^59^. However, we used a moderate, rather than an overly restricted, plane of nutrition in our model. In fact, we have previously shown that this rearing management protocol does not adversely affect the health or immune status of calves when compared to contemporaries fed to appetite^60^. Thus, the hypothesis that the immune system could act in the remodelling and/or development of the hypothalamus in our model, rather than to facilitate neuroprotection in response to diet restriction, is more likely. This is further supported by the fact that DEGs found in the ARC and the remaining hypothalamic tissue here were mostly involved in functional processes related to development, cellular growth and proliferation, molecular transport, metabolism and cell signaling. All together, these results suggest that the immune system, and *IL1B* in particular*,* could play an important role in the diet-dependent maturation process of the hypothalamus during the prepubertal period in cattle by regulating GnRH release. In addition, immune system genes could play a pivotal role aiding in the remodelling and/or developmental processes in the hypothalamus.

In line with our hypothesis, functional analysis revealed that a rapid BW gain during early calfhood induces differential expression of biochemical pathways consistent with earlier sexual development. Organ morphology, reproductive system development and function, developmental disorder were amongst the seven networks derived from the list of DEGs in the ARC. Interestingly, *CGA*, the most upregulated DEG in the ARC and a potential upstream regulator in this brain region, was one of the most connected genes within this network. *CGA* encodes for the alpha subunit of the glycoprotein hormones FSH, LH, and TSH. Despite the fact that CGA is a component part of three major hormones, the regulation of its expression has been poorly explored. In cattle, *CGA* has been identified in the amygdala, dorsal and ventral hypothalamus, and pituitary^61^. A study investigating the association between the variation in the expression of oestrous behaviour in dairy cows with the variation in gene expression in several brain areas reported that *CGA* was more highly expressed in the hypothalamus around midcycle (Day 12) compared with the start of oestrus in those cows with clear expression of oestrous behaviour^62^. The increase in *CGA* expression in the ARC of HI compared with MOD calves in our study could correspond with a preparation for an increase in gonadotropin before the onset of puberty. Another possible explanation for this fact could be the major use of *CGA* to produce FSH in MOD calves. As we have previously reported, calves on the moderate diet had higher FSH concentrations than HI calves at 10, 15, and 20 weeks of age^26^.

While the previously described methodologies allow for identification of genes that were differentially expressed in the ARC and the remaining hypothalamus of heifer calves fed with different nutritional plane diets, it does not allow for an evaluation of all genes expressed in these tissues nor do they provide information on the interaction of genes. Thus, we also carried out gene co-expression network analysis to identify key genes associated with early onset of puberty and their functions. Co-expression network analysis is a systems biology method for describing correlation patterns of genes across datasets, resulting in the formation of networks or clusters of highly correlated genes which may contribute to the expression of a particular trait^63^. In addition, hub genes, the most interconnected genes and, therefore, important in regulating the expression of several other genes within a network, may be identified. Cánovas et al.^46^ has previously utilised such an approach for the identification of genes governing puberty in heifers.

A high plane of nutrition increased *POMC* mRNA expression in the ARC. As discussed above, the *POMC* gene expressed in POMC/CART neurons encodes several peptides, including the anorexigenic α-MSH, which has excitatory effects on GnRH and kisspeptin neurons^25^. In the present study, WGCNA identified *POMC* as a hub gene regulating the expression of a further 319 genes. *POMC* was co-expressed and interacting with genes involved in GnRH signalling including adiponectin receptor 2-*ADIPOR2*^64^ and G protein subunit alpha I1-*GNAI1*^65^. Within the *POMC* network the most enriched molecular function was GTPase activator activity, which has been reported amongst the most enriched GO terms of DEGs between post- and prepubertal heifers by other authors^46^. Higher *POMC* expression was consistent with the increased systemic concentrations of leptin in heifers gaining weight at high rates observed for Allen et al.^24^, Alves et al.^38^, and Cardoso et al.^25^. Thus, in agreement with Cardoso et al.^25^, the POMC pathway may be important in mediating the nutritional acceleration of puberty in heifers. *PENK* is also a hub gene in the red cluster in the ARC that was not associated with any other cluster in the remaining hypothalamus. However, this gene was differentially expressed in the remainder of the hypothalamus but not in the ARC in this study, highlighting the importance of WGCNA to detect key genes involved in the regulation of networks that are not differentially expressed in a particular tissue. Interestingly, *PENK* maps to a region on bovine chromosome 14, which has been associated with fertility traits in cattle^66–68^. Similar to POMC, the *PENK* gene encodes an opioid precursor involved in neuron stimulation, highlighting a pivotal role of the opioid signalling pathway in the regulation of the transition through puberty in cattle. Additionally, we also identified *CHGA* as a hub gene in the remaining hypothalamus dataset, regulating the expression of a further 596 genes. The protein encoded by this gene is a member of the chromogranin/secretogranin family of neuroendocrine secretory proteins and is a precursor of three biologically active peptides (vasostatin, pancreastatin, and parastatin) and has also previously been implicated in pubertal development in heifers^27, 28^. These peptides act as autocrine or paracrine negative modulators of the neuroendocrine system. Secretogranins are involved in the packaging of peptide hormones and neuropeptides into secretory vesicles for uptake in target cells. Lastly, *CBLN2*, encoding cerebelin 2 precursor, which DeAtley et al.^69^ identified as important to puberty attainment in heifers was identified to be co-expressed with genes involved in insulin signalling (*INPPL1*, *MAP2K1* and *PRKAR1B*), highlighting the link between metabolic status and pubertal regulation. All together co-expression results from our study further suggest the importance of the findings observed by DeAtley et al.^69^ in which most of the differentially expressed peptides observed between pre- and postpubertal heifers were annotated with peptide packaging and processing functions (i.e., chromogranin-secretogranin family) as well as neuron stimulatory factors (*PENK*, *POMC* and *CBLN2* encoded proteins).

In conclusion, the essential maturation of the reproductive neuroendocrine system to support the onset of puberty relies, principally, on the appropriate development of the hypothalamus. Findings indicate that the functionality of each constituent of the HPG axis is sensitive to prevailing nutritional status though their respective rate of maturation does not occur in synchrony. Accelerated rates of body weight gain during early calfhood may facilitate pubertal development by programming hypothalamic centres that underlie the pubertal process, as early as 21 weeks of age. Metabolic signalling molecules such as leptin, insulin, and IGF1 are the proposed mediators of this process. Likely, the immune system, and IL1B particularly*,* could play an important role in the diet-dependent maturation process of the hypothalamus by regulating GnRH release. In addition, immune system genes could aid in the remodelling and/or developmental processes in the hypothalamus. Lastly, co-expression analysis identified novel genes involved in the control of hypothalamic maturation prior to the onset of puberty, highlighting the role of peptide-encoded genes involved in packaging and processing functions and neuron stimulation.

As discussed above, many studies have focused on nutritional changes and their impact on age at puberty. However, this is the first study clearly showing that transcriptomic changes in the ARC and the remaining hypothalamic tissue of heifer calves, as early as 21 weeks of age, are associated with prevailing plane of nutrition. These data can be further exploited to identify key genes central to orchestrating earlier sexual maturation in the heifer calf, as well as providing a basis for the better design and implementation of novel nutritional regimens to promote earlier onset of puberty.

## Methods

All experimental procedures involving animals were sanctioned by the Teagasc Animal Ethics Committee and were licensed by the Health Products Regulatory Authority, Ireland, in accordance with Statutory Instrument No. 543 of 2012 (under Directive 2010/63/EU on the Protection of Animals used for Scientific Purposes). In addition, the reporting in the manuscript follows the recommendations in the ARRIVE guidelines^70^.

### Calf management and sample collection

Angus X Holstein–Friesian heifer calves (n = 40) acquired from commercial Irish farms were enrolled in this study. All procedures were conducted at the Teagasc Grange Animal Bioscience Research Centre. Calves (19 ± 5 d of age; 51.2 ± 7.8 kg of BW, mean ± SD) were blocked by age, BW, sire, and farm of origin and randomly offered a high (HI, n = 14) or moderate (MOD, n = 15) plane of nutrition from 3 to 21 weeks of age to achieve a target growth rate of 1.2 kg/d and 0.5 kg/d, respectively based on recommendations from the National Research Council^71^. Full details regarding pre-weaning milk feeding plan and post-weaning feeding plan have been previously described^26^. Briefly, pre-weaned calves were individually fed milk replacer and concentrate in pelleted form using an electronic feeding system (Vario, Foster-Tecknik, Engen, Germany) and thereafter were penned according to treatment until 21 weeks of age. Post-weaning HI calves were fed concentrate *ad libitum* and MOD calves received 1 kg of concentrate daily. Hay was provided as a source of roughage and calves had ad libitum access to water. Calves were weighed using a calibrated scales (Tru-Test XR3000, load bars XHD 10,000, Auckland, New Zealand) on a weekly basis throughout the duration of the experiment. Of note, we have previously shown that neither rearing management protocol employed here adversely affects the health or immune status of calves^60^.

After 21 weeks (145 ± 3 d of age), all calves were euthanized using a lethal dose of phenobarbital administered intravenously. The brain was subsequently removed and the hypothalamus was isolated. Then, the ARC region was separated from the remainder of the hypothalamus according to Komatsu et al.^72^ and both sections were immediately snap frozen in liquid N_2_ and stored at -80° C pending analysis.

### RNA isolation and RNA sequencing

From the 40 calves, enrolled on the study, we selected a representative group for RNA analysis from both treatments, including only healthy calves within 0.5 SD units of the mean average daily gain for the group. Thus, total RNA was isolated from 29 hypothalamic and ARC samples (HI, n = 14; MOD, n = 15) using the Qiagen RNeasy Plus Universal kit (Qiagen, Manchester, UK) in accordance with the manufacturer’s instructions. Following isolation, RNA samples were quantified on the Nanodrop 1000 spectrometer (ThermoFisher Scientific, Dublin, Ireland), and RNA quality was assessed on the Agilent Bioanalyzer (Agilent Technologies, Cork, Ireland) using the RNA 6000 Bioanalyzer Nano Lab chip kit (Agilent), ensuring all samples were of high quality yielding RIN (RNA integrity number) values of greater than 8. Preparation of cDNA libraries for RNA-Seq as well as sequencing were undertaken commercially by Macrogen (Seoul, Korea). Libraries were prepared using the Illumina TruSeq Stranded mRNA kit (Illumina, San Diego, USA) with sequencing subsequently undertaken on an Illumina NovaSeq platform (Illumina). mRNA sequencing generated paired end reads of 150 base pairs in length.

### Bioinformatic and pathway analyses

Resultant reads following RNA-Seq were first checked for quality using Fastqc (version 0.11.7). Indexing adapters used for sequencing were subsequently removed using Cutadapt software (version 2.6); additionally, any low quality reads were also removed using this software. Trimmed sequencing reads were then aligned to the bovine reference genome (UMD3.1) using the Spliced Transcripts Alignment to a Reference (STAR) aligner (version 2.5.2.b). The quantmode function within STAR was utilised in order to quantify the number of sequencing reads aligned to each gene. Differential expression of genes was determining using the R (version 3.4.2 2017-09-28) Bioconductor package EdgeR (version 3.20.9^73^). Lowly expressed genes, any gene with less than one count per million in at least fourteen of the samples, were removed from the analysis and the data were then normalised using the trimmed mean of M-values normalisation method^74^. Exact tests were used for the detection of differentially expressed genes (DEGs) between calves on the HI diet and those on the MOD diet. Genes with a Benjamini–Hochberg false discovery rate (FDR) of 10% were considered differentially expressed. A fold change cut-off of > 1.5 was used for all DEGs. The resultant list of DEGs was then submitted to Ingenuity pathway analysis (IPA; Qiagen) in order to assign biological annotation. Within IPA, Fisher’s exact test was used with the Benjamini–Hochberg correction for multiple testing for the identification of over-represented pathways and over-represented molecular and cellular functions with a FDR of 10%, from DEGs between HI and MOD calves.

Gene co-expression networks were individually generated for the RNA-Seq transcriptome datasets from the ARC and the remainder of the hypothalamus using the Weighted Gene Co-expression Network Analysis (WGCNA) software package^63^. For this, each RNA-Seq dataset were separately filtered for lowly expressed genes and subsequently normalised in EdgeR as outlined above. Normalised count data were then Log_2_(x + 1) transformed in R. The WGCNA automatic network construction and module detection method was utilised to generate unsigned co-expressed gene networks. For each separate analysis, pair-wise weighted Pearson correlations were calculated between all pairs of genes in each dataset. Adjacency matrices were calculated to reach scale-free topology of the network (R^2^ > 0.09) by raising the co-expression matrix to a soft-threshold power of 20 for each dataset analysed. Following this, the topology overlap matrix was calculated, providing information on the similarly of the co-expression between two genes with all other genes in the network. Average linkage hierarchical clustering was then applied to the topology overlap matrix for each dataset resulting in the grouping of modules of co-expressed genes. Final modules of co-expressed genes generated were assigned colour identifiers to distinguish individual modules of co-expressed genes. Modules of co-expressed genes were then mined for genes that have previously been implicated in puberty development in heifers^27, 28^, as well as DEGs from the current study in order to provide biological information on the molecular interaction of these genes in puberty attainment.

## Supplementary Information

Supplementary Information.

## Data Availability

RNA-Seq data derived from the current study have been deposited within NCBI’s Gene Expression Omnibus and are available through accession IDs GSE153495 and GSE153498 for hypothalamus and arcuate nucleus datasets, respectively.
